# Seasonal malaria chemoprevention combined with community case management of malaria in children under 10 years of age, over 5 months, in south-east Senegal: A cluster-randomised trial

**DOI:** 10.1371/journal.pmed.1002762

**Published:** 2019-03-13

**Authors:** Jean Louis A. Ndiaye, Youssoupha Ndiaye, Mamadou S. Ba, Babacar Faye, Maguette Ndiaye, Amadou Seck, Roger Tine, Pape Moussa Thior, Sharanjeet Atwal, Khalid Beshir, Colin Sutherland, Oumar Gaye, Paul Milligan

**Affiliations:** 1 University Cheikh Anta Diop, Dakar, Senegal; 2 Ministry of Health and Social Affairs, Dakar, Senegal; 3 London School of Hygiene & Tropical Medicine, London, United Kingdom; Mahidol-Oxford Tropical Medicine Research Unit, THAILAND

## Abstract

**Background:**

Seasonal malaria chemoprevention (SMC) is recommended in the Sahel region of Africa for children under 5 years of age, for up to 4 months of the year. It may be appropriate to include older children, and to provide protection for more than 4 months. We evaluated the effectiveness of SMC using sulfadoxine-pyrimethamine plus amodiaquine given over 5 months to children under 10 years of age in Saraya district in south-east Senegal in 2011.

**Methods and findings:**

Twenty-four villages, including 2,301 children aged 3–59 months and 2,245 aged 5–9 years, were randomised to receive SMC with community case management (CCM) (SMC villages) or CCM alone (control villages). In all villages, community health workers (CHWs) were trained to treat malaria cases with artemisinin combination therapy after testing with a rapid diagnostic test (RDT). In SMC villages, CHWs administered SMC to children aged 3 months to 9 years once a month for 5 months. The study was conducted from 27 July to 31 December 2011. The primary outcome was malaria (fever or history of fever with a positive RDT). The prevalence of anaemia and parasitaemia was measured in a survey at the end of the transmission season. Molecular markers associated with resistance to SMC drugs were analysed in samples from incident malaria cases and from children with parasitaemia in the survey. SMC was well tolerated with no serious adverse reactions. There were 1,472 RDT-confirmed malaria cases in the control villages and 270 in the SMC villages. Among children under 5 years of age, the rate difference was 110.8/1,000/month (95% CI 64.7, 156.8; *p <* 0.001) and among children 5–9 years of age, 101.3/1,000/month (95% CI 66.7, 136.0; *p <* 0.001). The mean haemoglobin concentration at the end of the transmission season was higher in SMC than control villages, by 6.5 g/l (95% CI 2.0, 11; *p =* 0.007) among children under 5 years of age, and by 5.2 g/l (95% CI 0.4, 9.9; *p =* 0.035) among children 5–9 years of age. The prevalence of parasitaemia was 18% in children under 5 years of age and 25% in children 5–9 years of age in the control villages, and 5.7% and 5.8%, respectively, in these 2 age groups in the SMC villages, with prevalence differences of 12.5% (95% CI 6.8%, 18.2%; *p <* 0.001) in children under 5 years of age and 19.3% (95% CI 8.3%, 30.2%; *p <* 0.001) in children 5–9 years of age. The *pfdhps*-540E mutation associated with clinical resistance to sulfadoxine-pyrimethamine was found in 0.8% of samples from malaria cases but not in the final survey. Twelve children died in the control group and 14 in the SMC group, a rate difference of 0.096/1,000 child-months (95% CI 0.99, 1.18; *p =* 0.895). Limitations of this study include that we were not able to obtain blood smears for microscopy for all suspected malaria cases, such that we had to rely on RDTs for confirmation, which may have included false positives.

**Conclusions:**

In this study SMC for children under 10 years of age given over 5 months was feasible, well tolerated, and effective in preventing malaria episodes, and reduced the prevalence of parasitaemia and anaemia. SMC with CCM achieved high coverage and ensured children with malaria were promptly treated with artemether-lumefantrine.

**Trial registration:**

www.clinicaltrials.gov NCT01449045.

## Abstract 2

Jean Louis Ndiaye & colleagues assess seasonal malaria chemoprevention added to community case management in children younger than 10 years of age in this cluster-randomized trial in southern Senegal

## Author summary

**Why was this study done?:**

- Seasonal malaria chemoprevention (SMC) is recommended for children under 5 years of age for up to 4 months of the year in the Sahel and sub-Sahel, but could be useful in older children and in areas with a longer transmission season.
- This study was done in south-east Senegal—where the main malaria transmission season lasts for 5 months and there is a high burden of malaria in the under-10 age group—to determine the effectiveness of SMC delivered for 5 months to children under 10 years of age by community health workers (CHWs) who were also providing community case management for malaria.

**What did the researchers do and find?:**

- Twenty-four villages that had a resident CHW providing community case management for malaria were randomised: In 12 of the villages the CHW was trained to administer SMC once a month for 5 months during the transmission season to all children aged between 3 months and 10 years, while the other 12 villages did not have SMC.
- Malaria cases confirmed by rapid diagnostic test (RDT) were recorded by the CHWs. At the end of the transmission season, children were surveyed to measure their haemoglobin concentration and to check for the presence of malaria parasites.
- SMC was associated with 111 fewer malaria cases per 1,000 children per month in children under 5 years of age and 101 fewer cases per 1,000 children per month in children aged 5–9 years as compared with community case management alone.
- SMC was associated with a reduction in the percentage of children (in both age groups) with anaemia at the end of the transmission season of 18%, and a reduction in the percentage of children with malaria parasitaemia of 73%, compared to community case management alone.
- The study did not find a reduction in mortality and did not measure the impact of SMC on severe malaria.

**What do these findings mean?:**

- SMC can be administered effectively for 5 months in children up to the age of 10 years, the treatments are well tolerated, and high coverage can be achieved.
- Twelve countries now have SMC programmes, with SMC provided for 3 or 4 months to children under 5 years of age (with the exception of Senegal, where children under 10 years are included). The findings of this study indicate that SMC could be administered over a longer period and/or to a wider age group. The strategy could be adapted depending on local epidemiology, to increase the impact on malaria.

## Introduction

**Fig 1:**
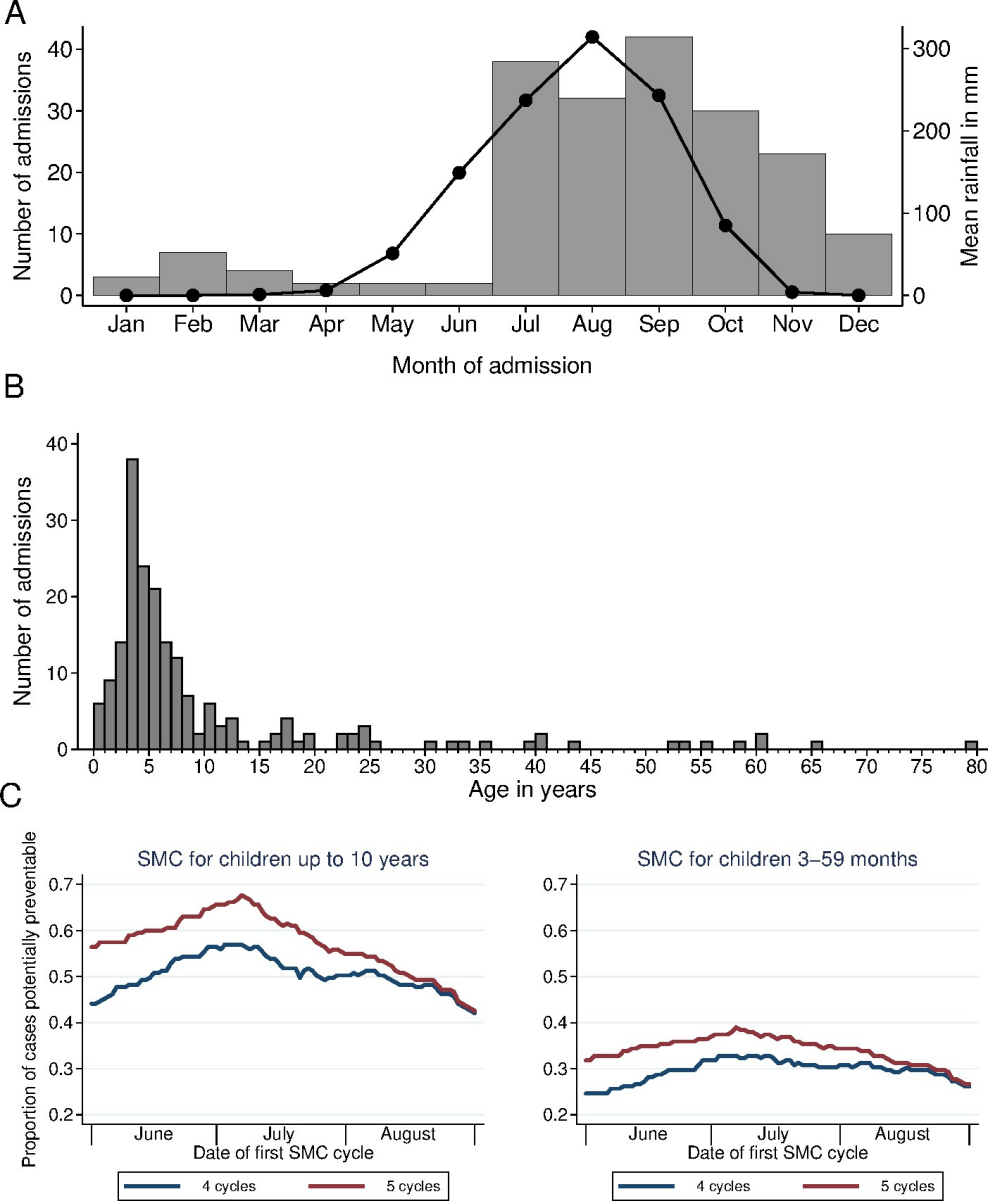
The age distribution and seasonal pattern of patient admissions to Saraya Hospital with malaria in 2011. The number of patients admitted to Saraya Hospital with severe malaria in 2011, by month of admission (A) and by age (B). Individual patient data (age, date of admission, and primary diagnosis) were extracted from the hospital registers for all patients admitted from 1 January to 31 December 2011. (A) also shows a line indicating monthly rainfall (on the right-hand axis). (C) shows the proportion of cases that could potentially have been prevented by seasonal malaria chemoprevention (SMC), if SMC had been provided for children up to the age of 5 years or up to the age of 10 years, for 4 consecutive months or for 5 consecutive months starting on the date indicated. The *y*-axis shows the cases in 2011 in the given age group that fell in a 4- or 5-month window starting from the given date, as a proportion of total cases. From January to December there were a total of 195 admissions with severe malaria, 147 of them under 10 years of age, and of those, 91/147 (62%) were under 5 years and 56 (38%) were 5–9 years of age. In all, 7/195 patients died, 1 aged 18 years and the other 6 aged under 5 years. Ten of the 195 patients had cerebral malaria; all these patients were under 10 years of age—7 of them under 5 years of age and 3 aged 5–9 years. In all, 165 (85%) of the 195 cases occurred in the 5 months from July to November (rainfall data from Climate-Data.org).

Seasonal malaria chemoprevention (SMC), the administration once per month of antimalarial treatment using sulfadoxine-pyrimethamine (SP) plus amodiaquine (AQ) to prevent malaria, is currently recommended in the Sahel region of Africa for children under 5 years of age, for up to 4 months of the year [1]. In many of parts of this region, it may be appropriate to consider extending the scope of SMC programmes by including older children [2–6] and, in areas with a longer transmission season, by providing protection for more than 4 months each year. In south-east Senegal, for example, individual patient data from Saraya Hospital extracted from registers for the year 2011 showed that 75% of cases admitted to hospital with severe malaria were children under 10 years of age, and 85% of the severe malaria cases occurred over the 5 months from July to November (Fig 1). In addition, in remote areas, it may be beneficial to combine SMC with community case management (CCM) [7] or to provide SMC teams with access to rapid diagnostic tests (RDTs) and artemisinin combination therapies (ACTs) so that children who are unwell can be screened quickly and treated with rapid-acting ACTs if they are shown to have malaria.

To guide SMC policy in Senegal, we undertook a cluster randomised trial to evaluate the effectiveness of SMC in children under 10 years of age, extended over 5 months, in a high-transmission area in the south-east of the country. The study was done in villages where CCM for malaria (PECADOM, *prise en charge à domicile*) had been established, in order to assess the added benefit of combining SMC with PECADOM and the feasibility of both interventions being delivered together.

## Methods

### Study site

**Fig 2:**
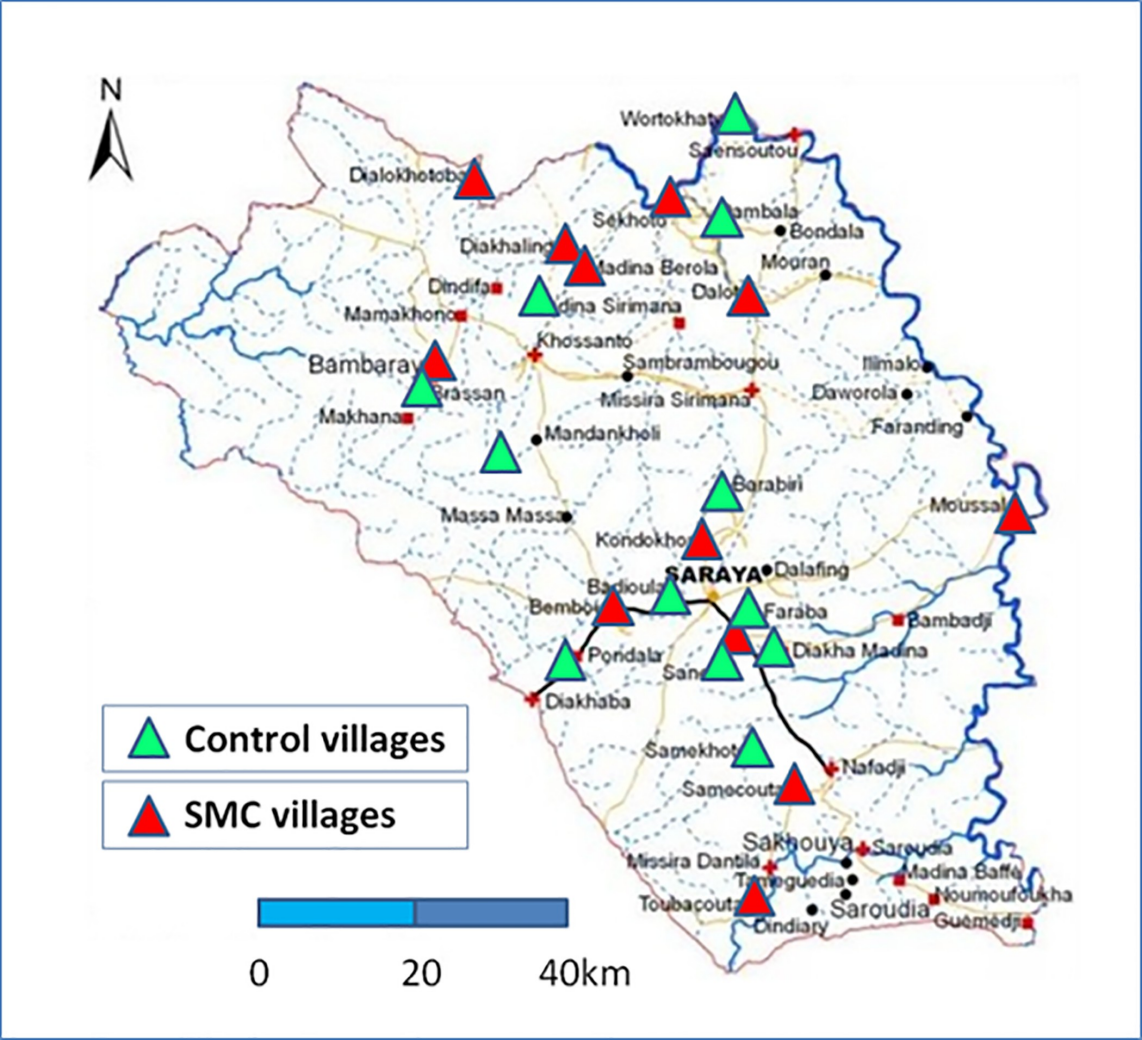
Map of the study area showing the location of the study villages. SMC, seasonal malaria chemoprevention.

Saraya Department (district, Kedougou Region), in the south-east of Senegal, bordering Mali to the east and Guinea to the south, is composed of the rural communities of Sabadola, Khossanto, Bembou, Missirah Sirimana, and Medina Baffe, and includes 102 villages occupying a land area of 7,803 km^2^, with an estimated total population of 37,048 (2009 estimate). Over 70% of the population live more than 15 km from a health facility. The main ethnic groups are Malinke and Diakhanke. The climate is Sudano-Sahelian with 2 seasons, a dry season from November to May and a rainy season from May to November. Mean daily temperatures range from 14°C to 36°C from July to February and 21°C to 40°C from March to June. The malaria transmission season is longer than in other parts of Senegal, lasting about 6 months. This study took place in 24 rural villages in Saraya Department where PECADOM had been established in 2009–2010: 12 villages where malaria case management was provided (for patients of all ages) by a village volunteer (*distributeur de soins à domicile* [DSDOM]) who can test for malaria with an RDT and treat with artemether-lumefantrine, and 12 villages with a health hut (*case de santé*) managed by an *agent de santé communautaire* (ASC), equipped with RDTs, artemether-lumefantrine, oral rehydration solution, zinc, and an antibiotic (amoxicillin), who can treat diarrhoea, respiratory infection, and malaria. (In this paper we also use the term community health worker [CHW] to refer to both DSDOMs and ASCs.) The study population comprised all children aged from 3 months to 119 months living in these villages. A map of the study area is shown in Fig 2.

### Study design

Twenty-four villages, 12 where malaria case management was provided by a DSDOM and 12 with a *case de santé* managed by an ASC, were selected, forming 2 strata for randomisation and analysis. A census of the study villages was undertaken in July 2011 by the 24 CHWs and 10 nurses from the health posts serving the area who were trained for this purpose. Children who would be aged 3–119 months at the time scheduled for the first SMC treatment and who had no known allergy to SMC drugs were eligible to participate. Cluster randomisation was chosen to allow the overall impact of SMC, including any indirect effect on transmission, to be captured, and to allow assessment of the impact of adding SMC on the overall workload of the health workers. Randomisation in each stratum was constrained to ensure balanced allocation with respect to population size, distance to the nearest health post or hospital, and the malaria incidence in the village the year before the trial, and was performed using Stata version 11 (StataCorp, College Station, Texas) by a statistician at the London School of Hygiene & Tropical Medicine. The number of permutations of 12 clusters in each of 2 strata is [12!/(6!6!)]^2^ = 853,776. One permutation was chosen from a total of 229 permutations that met the requirement that for each of the variables within each stratum the difference in the mean between the 2 groups should be less than 15% of the overall mean for that variable. The primary endpoint was malaria defined as fever (axillary temperature ≥ 37.5°C) or a history of fever, with no obvious other cause of the fever, and a positive result for malaria by RDT. It was intended to use microscopically confirmed cases for the primary analysis, but although CHWs were trained to make blood films, it was not possible to obtain blood films for all suspected cases. RDT-confirmed malaria was therefore used for the primary analysis. The secondary endpoints were coverage of SMC; parasitaemia, gametocyte carriage, haemoglobin (Hb) concentration, anaemia (Hb < 110 g/l), and severe anaemia (Hb < 60 g/l) at the end of the transmission season; the presence of molecular markers of resistance to SP and AQ in samples from malaria cases and in samples taken in the survey of children at the end of the transmission season; mortality (any cause); and occurrence of adverse events at any time during the study period and within 10 days of each SMC administration, with an emphasis on skin rash, diarrhoea, vomiting, and signs of jaundice/liver disease. Process indicators included the number of courses of SP and AQ administered, the number of children treated each month, the number of person-days spent by health workers on SMC delivery, the number of supervision visits made to the villages, the number of training sessions held, and the number of persons trained; these data will be published in a separate report.

### Ethics

Members of the study team held meetings with community, administrative, and religious leaders to explain the aims and activities of the study and to seek community approval and promote community ownership of the programme. Staff then visited each household to explain the study in the local language, provided an information sheet, and sought signed consent from parents. Children whose parents consented were enrolled, and mothers/caregivers were issued with an ID card bearing the details and study ID number for each eligible child in their care. The ID card was used to identify children if malaria was diagnosed and, for those in clusters randomised to receive SMC, to document SMC courses of treatment received. A data and safety monitoring board was convened to oversee the project and review data on tolerability and safety. The study was approved by the Comité National d’Ethique pour la Recherche en Santé of Senegal. The National Director of Health gave administrative authorisation to conduct the study. Trial registration (ClinicalTrials.gov NCT01449045) was started on 5 July 2011, but due to an administrative oversight was not completed until 11 September 2011; the trial was posted on the ClinicalTrials.gov website on 6 October 2011.

### Malaria surveillance

In each village, the PECADOM system supports a resident CHW to provide malaria case management. Persons with fever are encouraged to attend the health hut or the home of the health worker to be tested with an RDT (Core Malaria Pf test, CORE Diagnostics, New Delhi, India) and treated with artemether-lumefantrine if the test is positive. Consultations are recorded in a register to document the test results and treatment given. Severe cases, children less than 2 months of age, and pregnant women are referred to the health post or district hospital. For this study, refresher training in CHW roles was provided, and, in addition, the CHWs were trained to prepare thick and thin blood films, which were collected by the trial team for microscopic examination to crosscheck the RDT results. CHWs were also trained to take blood spots onto filter paper for molecular analysis, and to record and report any adverse events. Nurses at health posts and district hospitals were also trained to prepare thick and thin films.

If a child was unwell on the day of the SMC visit, caregivers were asked to bring the child to the health hut or home of the DSDOM for testing with malaria RDT; those who tested positive were treated with artemether-lumefantrine, and those who tested negative received SMC and were then referred to the nearest health post.

Participants who presented at health posts or at the district hospital with fever were similarly tested with an RDT and had thick and thin blood films taken by the nurse, which were read later. Treatment of study participants seen at the health posts for other conditions was carried out in accordance with national guidelines.

All positive RDTs were retained for subsequent DNA extraction.

Registers for recording SMC administration (in SMC villages) and morbidity (in all study villages), with a list of all children enrolled in each village, were made available for each CHW. In SMC villages, the dose of SP and AQ administered to each child was recorded in the register. All consultations for illness were also recorded in the registers (including date, symptoms, RDT result, and treatment). In addition, in each health post, consultation records were reviewed by the nurse to identify all consultations of children from the study villages.

### Mortality surveillance

A reporter was appointed in each village to record deaths in children aged less than 10 years. A verbal autopsy was performed by project staff using a questionnaire adapted from WHO and the INDEPTH Network for children under the age of 12 years. Two physicians reviewed the reports and coded the cause of death independently; any discrepancies were resolved by a panel. In addition, in SMC villages, some deaths that had not been reported were detected when the study team investigated children who were absent from SMC cycles.

### Community sensitisation

Social mobilisation was organised through local meetings before the start of SMC administration to explain when the campaign would start, how the treatments would be administered, and the importance of adherence to the drug regimen. Local community radio and announcements in the mosques were used to inform the community about the timing of SMC and to emphasise the importance of ensuring children received treatment each month and adhered to the regimen each month.

### SMC administration

Training for CHWs was organised over 3 days to explain how to administer SMC, including checking for fever and for any intake of SP or AQ or sulfa-containing antibiotics in the preceding month, checking the child’s age and selecting the correct dose, administering SP and the first dose of AQ, and explaining to the caregiver how to administer the remaining 2 doses of AQ. CHWs went door to door from 7 AM in the morning and in the evenings, when children and caregivers were most likely to be at home, starting cycles on Friday afternoon or Saturday morning so as to be able to reach school-age children. Infants aged 3 to 11 months received 0.5 tablets of SP and 3× 0.5 tablets of AQ, children aged 1 to 5 years received 1 tablet of SP + 3× 1 tablet of AQ, and children aged 6 to 9 years received 1.5 tablets of SP + 3× 1.5 tablets of AQ. This dosing schedule was selected to minimise under- and overdosing, based on weight-for-age data from a survey in Niakhar [3], to keep simple age categories, and to avoid the use of quarter tablets. Sweetened dispersible tablets of SP and AQ were used (SP: 500/25-mg tablets; AQ: 153-mg tablets, both manufactured by Kinapharma, Accra, Ghana), mixed with water. Household water and utensils were used for SMC administration. SMC administration was conducted once a month for 5 months starting in July 2011. Each SMC cycle was planned to last 3 to 5 days.

### Surveillance of adverse events

Caregivers of study children were encouraged to bring children to the CHW if they were unwell after SMC administration.

### Monitoring of adherence

A survey was conducted during the fourth and the fifth SMC cycles, to ask caregivers about adherence: 318 children were surveyed in 8 SMC villages during cycle 4, and 462 children in 3 SMC villages during cycle 5.

### Supervision of SMC administration

Project staff (4 local supervisors and 2 project coordinators) visited each village 2 weeks after each SMC cycle to collect data on SMC coverage, malaria cases, adverse events, and deaths; to collect blood slides; and also to check drug supplies in order to prepare for the next administration cycle.

### Baseline census

A census was conducted in July 2011 by the CHW in each village to ask about household assets (for assessment of socioeconomic status), level of education, and other characteristics of adults in each household, and to record ownership and condition of bednets.

### Survey of the prevalence of parasitaemia and anaemia

A survey was conducted in each village at the end of the transmission season, in December—1 month after the last SMC cycle—to take a finger prick blood sample to make blood films for measurement of *Plasmodium falciparum* parasitaemia and gametocyte carriage, to measure Hb concentration using a hemocue machine (Hb301, HemoCue, Ängelholm, Sweden), and to take blood spots on filter paper for molecular analysis of markers of resistance to SMC drugs. This survey included 35 children in each age group (aged less than 5 years and 5–9 years) in each village, by simple random sampling from the census list. A questionnaire was administered to assess use of insecticide-treated bednets by study children; caregivers’ knowledge, attitudes, and practices in relation to malaria; acceptability of SMC; and adherence to the unsupervised AQ doses in the last SMC cycle.

### Laboratory methods

Thick smears were stained with Giemsa stain and 200 high-power fields (HPFs) examined before a smear was declared negative. Parasite density was expressed per microlitre with the assumption that 1 parasite per HPF equals a density of 500 parasites per microlitre. Slides were read independently by 2 laboratory technicians, who were unaware of which village the slides came from. If there was disagreement between their readings on parasite positivity or if the difference in the log10 densities recorded was more than 1.5, slides were read by a third technician. Discrepancies occurred mainly in smears with very low parasite densities. A sample of tablets of SP and AQ were tested at the Drug Quality Control National Laboratory in Dakar at the beginning and at the end of the study for drug content and dissolution. At the end of the study, a further sample of AQ tablets from the field was tested for drug content at the national laboratory in Dakar; drug content was within 5% of the nominal level. For clinical cases, a sample of positive RDTs was selected (130/270 from SMC villages and 110/1,472 from control villages) and parasite DNA was extracted directly from the RDT cassette. For cross-sectional surveys, blood samples were collected on filter paper. For both sample types, DNA was extracted by the Chelex method [8] and mutations in the *pfmdr1* gene (codons 86 and 184), *pfdhfr* gene (codons 51, 59, and 108), and *pfdhps* gene (codons 431, 436, 437, 540, 581, and 613) were typed.

### Statistical methods

The study was designed to have at least 90% power to detect a reduction in the incidence of malaria of 50% assuming an incidence rate of 0.04 per child per transmission season (i.e., 8 per 1,000 child-months, based on data from 2009 from district health records showing there were 290 positive cases with fever or history of fever and a positive RDT among children under 5 years in the district August–December, with an estimated total population of children of 7,000). District health records from 2010 showed a higher rate of about 0.1 per child per month, or 0.5 per child per 5-month transmission season. We estimated that if 24 clusters were randomised and each cluster included approximately 220 children, and assuming a coefficient of variation of the incidence rate among clusters of 0.25, the trial would have 90% power to detect a 50% reduction in incidence associated with SMC at the lower incidence rate and 90% power for a 33% reduction at the higher rate.

The parasitological survey at the end of the transmission season was designed to have 80% power to detect a reduction in mean Hb of 5 g/l in each age group (<5 years and 5–9 years), and 80% power to detect a 40% reduction in parasite prevalence in both age groups combined. For mean Hb concentration, data from a study in 2004 in Niakhar [9] gave a pooled estimate of within-village standard deviation of 16 g/l, and an intra-class correlation of ρ = 0.04, in children less than 5 years of age. Standard calculations give a sample size of *N =* 334 for an individually randomised study to have 80% power to detect a difference of 5 g/l if the SD is 16 g/l. The number required, allowing for clustering, is *c*∙*N*∙(1 − roh)/(*c* − *N*∙ρ) for *c* clusters. This gives a total sample size of 667 in each age group, or 1,334 in total. Allowing for non-response, we therefore set the sample size at 70 children per village, 35 in each age group, to be selected by simple random sampling from the census list in each village. For the prevalence of parasitaemia, this sample size would give 80% power to detect a 40% reduction (both age groups combined) if the coefficient of variation between villages was 0.3 and the prevalence was 30% in the control arm. For adherence surveys, it was estimated that about 300 children were required to estimate the percentage adherence within ±6% if 80% of children adhered and assuming a design effect of 2.

There were 3 analysis sets: Analysis of malaria incidence was by intention to treat, including all children who were enrolled (all children in the correct age range who were present in the study area at the initial census and were issued with a malaria ID card, regardless of the number of treatments received; *n =* 4,546); analysis of survey outcomes included all children included in the end of season survey (*n =* 1,424); and adherence was assessed in 318 children surveyed after cycle 4 and 462 after cycle 5. The time at risk for analysis of malaria incidence started on 27 July 2011 (the first day of SMC) and ended on 31 December 2011, with observations censored at the date of death or emigration if the child was known to have died or permanently emigrated. The timing of malaria episodes was illustrated by plotting the Nelson–Aalen cumulative hazard (an estimate of the mean number of episodes per child) over time in each group. Efficacy (percent reduction in incidence associated with SMC) and the rate difference between the two study arms were calculated using a ratio estimator of the incidence rate in each group of the study, with stratification by village type (served by *case de santé* or DSDOM). Interaction with age was tested using a *t* test comparing between the study arms the within-cluster differences in the incidence rate between age groups, as described by Cheung et al. [10].

Effects of SMC on the prevalence of parasitaemia, gametocyte carriage, mean Hb concentration, and prevalence of anaemia (Hb < 110 g/l) and severe anaemia (Hb < 60 g/l) were estimated from the survey at the end of the transmission season using a survey ratio estimator (with the associated standard errors in each group) and testing for interaction based on *t* tests comparing—between trial arms—the between-age-group differences. The distribution of Hb concentration at the end of the transmission season in each arm of the trial was plotted using a kernel density estimator using the kdensity function of Stata (with the Epanechnikov kernel with a half-width of 6). Analyses were performed using Stata version 14 (StataCorp, College Station, Texas).

### Data management

Village registers that listed each child recorded in the census were printed for health workers to record SMC administration and consultations for illness. All consultations for illness were recorded, and the registers checked for completeness during weekly supervisory visits; at the end of the study, the data were entered into a database in Dakar. SMC administration was recorded in the registers, and similarly checked and entered. All data were entered into an SQL database using an Access (Microsoft) front end. The accuracy of data input by each clerk was monitored by checking a sample of data records, and all data entered by that clerk were re-entered if errors were found. Automated checks for consistency and range errors were done, and queries resolved before the dataset was locked for analysis.

## Results

**Fig 3:**
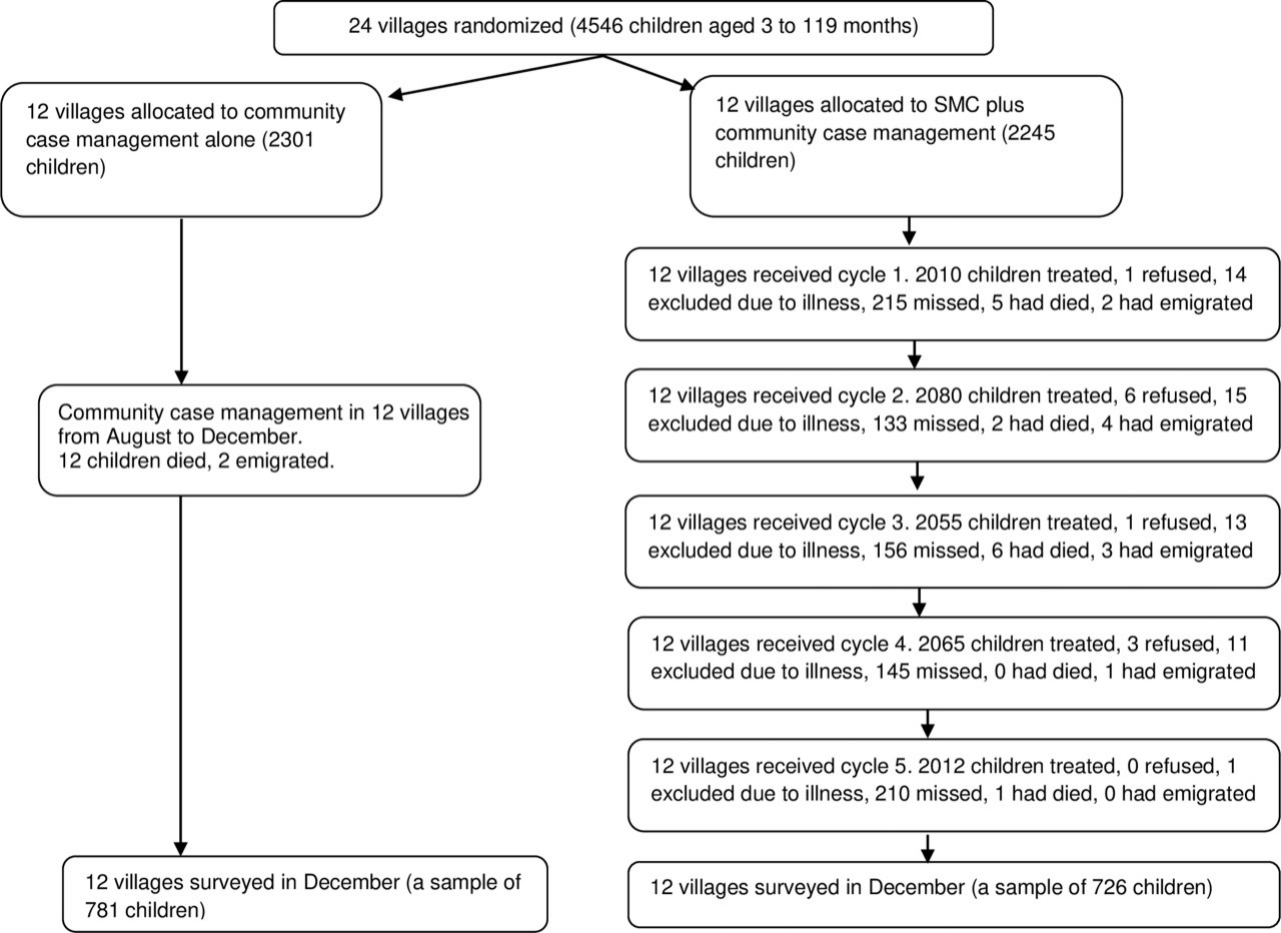
Trial profile. SMC, seasonal malaria chemoprevention.

Twenty-four villages were randomised to receive SMC in addition to CCM for malaria or CCM alone (Fig 3). The 2 groups were similar with respect to baseline characteristics (Table 1); the distance to the nearest health centre ranged from 4 km to 30 km, and insecticide-treated bednet coverage was high. From July to November in 2010, the year before the trial, from district health records there were a total of 1,238 cases of malaria in children under 5 years of age in the study villages, an incidence rate of 0.12 per child per month.

**Table 1:** Characteristics of the study villages at baseline.

Variable	CCM	SMC+CCM
Number of villages*	12	12
Total number of children aged 3–59 months	1,134	1,167
Total number of children aged 60–119 months	1,063	1,182
Mean (range) number of children aged 3–59 months per village	94.5 (39–239)	97.3 (29–183)
Mean (range) number of children aged 60–119 months per village	88.6 (30–257)	98.5 (28–205)
Mean (range) distance to the nearest health post or health centre	13 km (4–30)	17 km (5–21)
Percentage of children who slept under an ITN^#^	91%	94%
Mean malaria incidence/child/month the season before the trial^†^	0.12	0.11

*Six of the villages in each arm had CCM provided by a *distributeur de soins à domicile* (DSDOM), the other 6 had CCM provided by an *agent de santé communautaire* (ASC) based at a health hut.

^#^Aged <5 years who slept under an ITN the night before the survey was conducted in July 2011.

^†^From July to November 2010.

CCM, community case management; ITN, insecticide-treated net; SMC, seasonal malaria chemoprevention.

### SMC delivery

**Fig 4:**
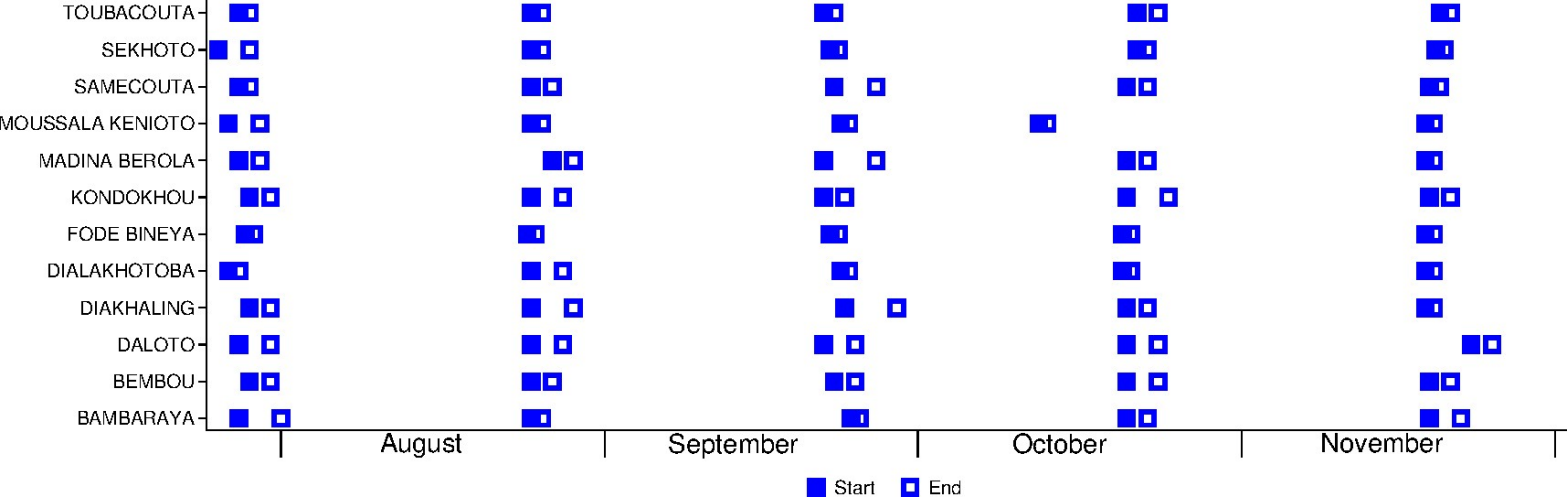
Timing of the 5 monthly cycles of seasonal malaria chemoprevention delivery in each village.

A total of 10,222 SMC treatments were administered over 5 months. The percentage of children treated each month was 89.8% (2,010/2,238) in cycle 1, 93.2% (2,080/2,232) in cycle 2, 92.4% (2,055/2,223) in cycle 3, 92.9% (2,065/2,222) in cycle 4, and 90.6% (2,012/2,221) in cycle 5 (Fig 3). On average, each month, 0.6% were excluded (due to illness or non-eligibility), and 0.1% refused. In villages with health huts, the mean number of children treated per day was 66, and the mean duration of SMC administration was 4 days per month. In villages with a DSDOM, the mean number of children treated per day was 43, and the mean duration of SMC administration was 3.7 days per month. Most villages adhered closely to the recommended interval of 28 days between cycles (Fig 4), with the exception of one village that started cycle 4 a week early and another village that started cycle 5 a few days late.

### Adherence

When adherence to the 3-day regimen was assessed in a sample of 318 children in October, caregivers reported that 99.4% of children who received SMC had taken the first day’s doses (SP and the first dose of AQ), 98.4% had taken the second daily dose of AQ, and 96.8% had taken the third dose of AQ. Adherence was assessed again in 462 children in November, and caregivers reported that drugs were administered to 462 (100%) on the first day, 455 (98%) on the second day, and 447 (97%) on the third day. When caregivers were asked if the child swallowed all of the medicine, caregivers reported that 116 (25%) children spat out some of the medicine administered on the first day, 43/455 (9.5%) spat out some of the medicine on the second day, and 34/447 (7.6%) on the third day. Three children started vomiting more than 1 hour after the dose on the second day. When caregivers were asked to show any leftover medication, 38 (8.2%) had some leftover tablets or half tablets on day 4.

### Incidence of malaria

**Fig 5:**
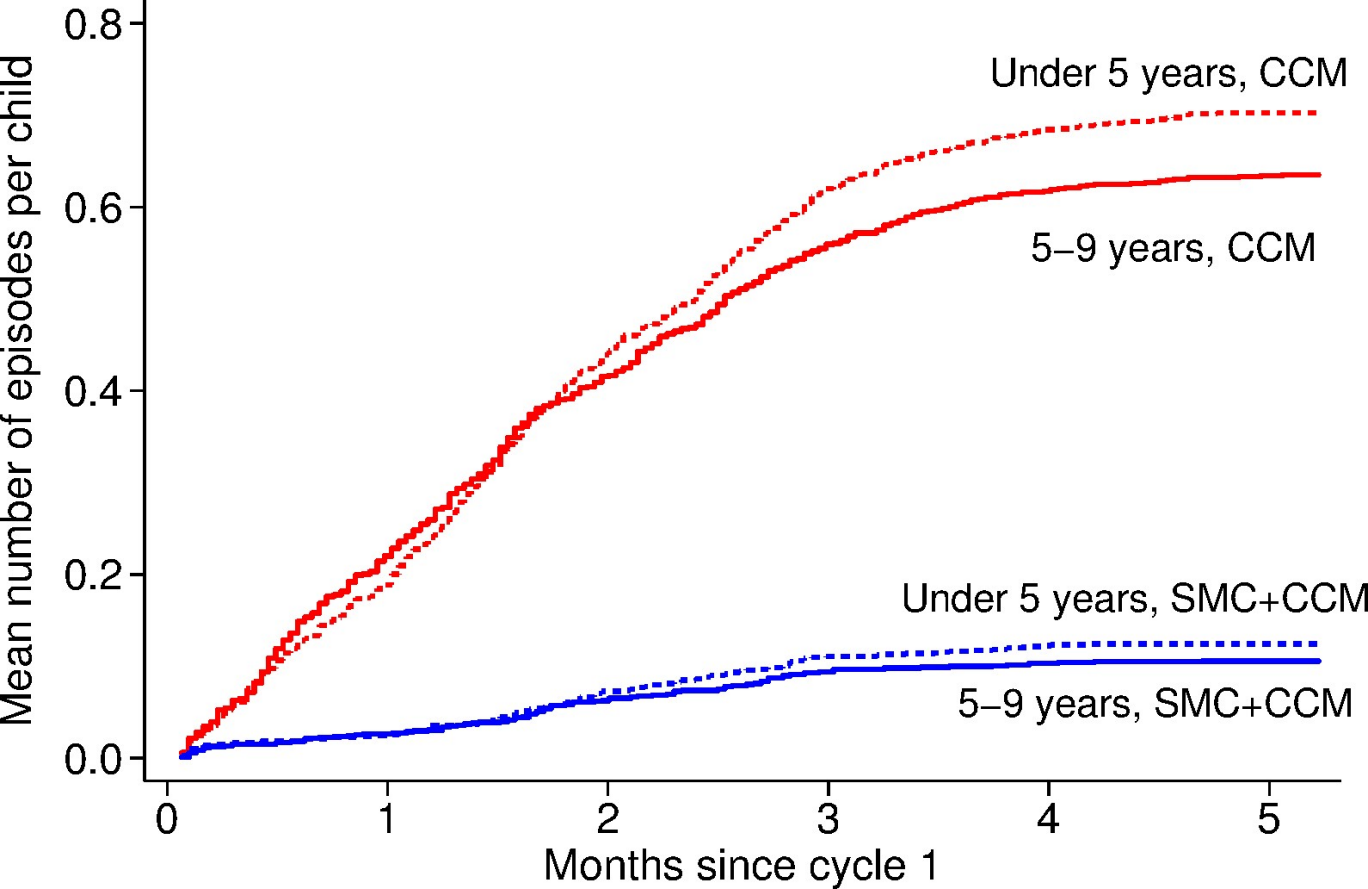
The timing of RDT-confirmed malaria episodes in each age group, in SMC and control villages, for 5 months from the SMC start date. The *y*-axis shows the Nelson–Aalen cumulative hazard, equal to the mean number of episodes of malaria experienced per child from the start of the study up to the given time (in months since the date when cycle 1 started in SMC villages), in children in villages targeted with SMC and in the control villages. CCM, community case management; RDT, rapid diagnostic test; SMC, seasonal malaria chemoprevention.

**Fig 6:**
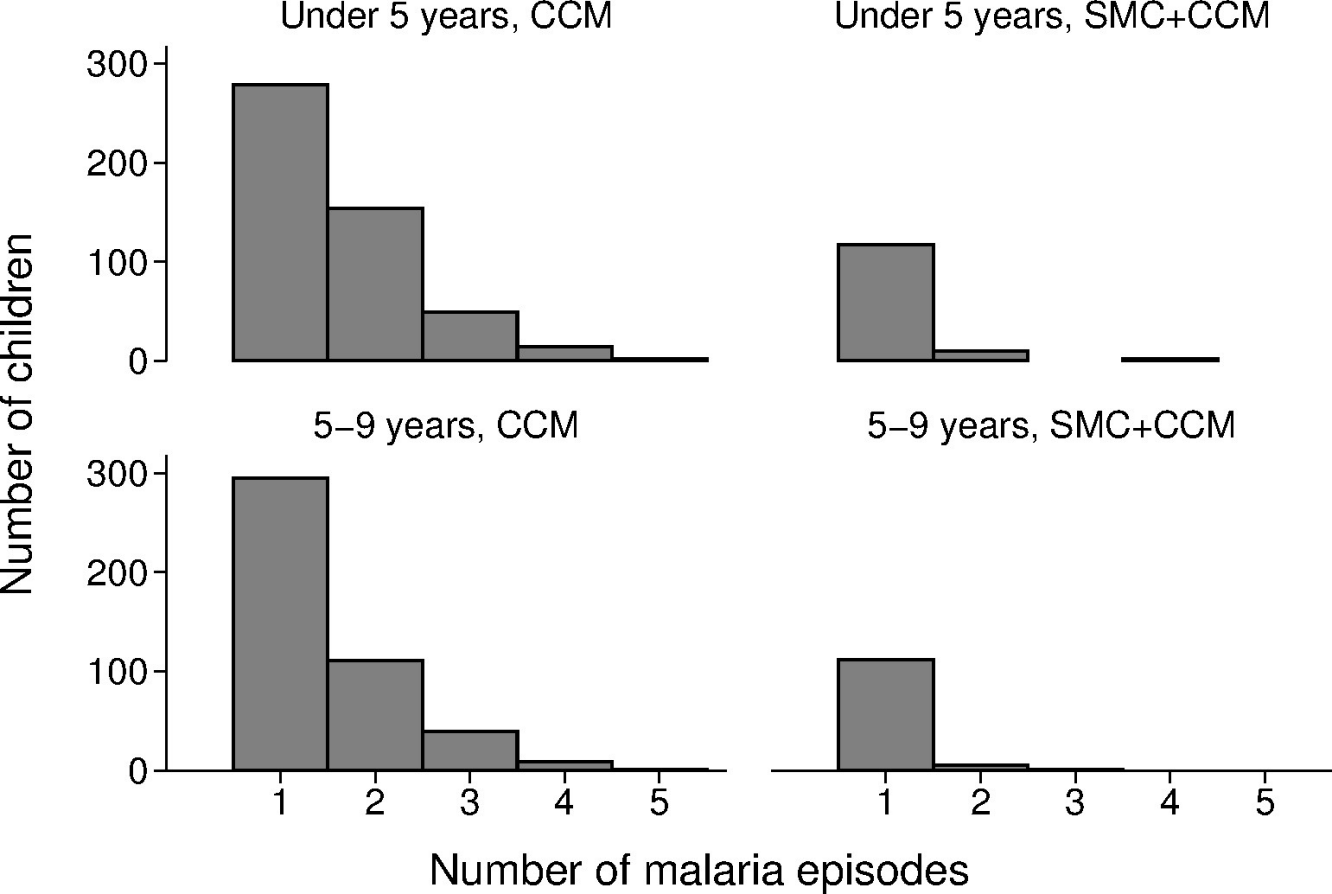
The effect of SMC on the number of children who experienced multiple episodes of malaria during the transmission season. The number of children who were treated for RDT-confirmed malaria 1, 2, 3, 4, or 5 times is shown for children in each age group, in villages targeted with SMC and in control villages. CCM, community case management; RDT, rapid diagnostic test; SMC, seasonal malaria chemoprevention.

A total of 1,742 cases of malaria confirmed by RDT were reported between the date cycle 1 started (27 July 2011) and 31 December 2011 in children less than 10 years of age—1,472 cases in the 12 villages with CCM alone and 270 cases in the villages with SMC, yielding incidence rates of 128.3 and 22.0/1,000/month, respectively (Table 2). In the control villages, 797/1,472 (54%) cases were in children who were less than 5 years of age (their age at cycle 1), and 675 (46%) were in children aged 5–9 years; the incidence rates were 134.6 and 121.6/1,000/month in each age group, respectively (Table 3). The number of episodes per child ranged from 0 to 5; the mean number of episodes per child was 0.67 (Fig 5). In all, 43% of children had malaria at least once, 17% had 2 or more episodes of malaria, and 3 children had 5 episodes of malaria (Fig 6). In the SMC group, 145/270 cases (54%) were in children aged less than5 years, and 46% were in children aged 5–9 years; the incidence rates were 23.8 and 20.2/1,000/month in each age group, respectively. The mean number of episodes per child was 0.11; 11% had malaria at least once, and 0.8% had 2 or more episodes of malaria. The efficacy of SMC was 83% (95% CI 74%, 89%) in both age groups combined. The malaria incidence rate difference between SMC and control villages was 110.8/1,000/month (95% CI 64.7, 156.8) in children under 5 years of age and 101.3 (95% CI 66.7, 136.0) in children 5–9 years of age, indicating that SMC was associated with a reduction of over 100 malaria cases per 1,000 children in each age group each month. The breakdown by calendar month (both age groups combined) is shown in Table 4. Efficacy was similar in each month, but the incidence and hence reduction in number of cases decreased, from about 180 cases reduced per 1,000 in each of the first 2 months, to a reduction of about 130 cases per 1,000 in the third month, 50 per 1,000 in the fourth month, and 16 per 1,000 in the fifth month.

**Table 2:** Efficacy and impact of SMC against malaria, parasitaemia, gametocyte carriage, anaemia, and deaths: Both age groups combined.

Outcome	CCM	SMC+CCM	Efficacy (95% CI)	Difference (95% CI)
Malaria cases, rate/1,000 (number of cases/1,000s of person-months)*	128.3 (1,472/11.48)	22.0 (270/12.27)	83% (74%, 89%)	106.3 (66.9, 145.6), *p <* 0.001
Prevalence of parasitaemia^†^, percent (number positive/number sampled)	21.5% (152/707)	5.7% (41/717)	73% (52%, 85%)	15.8% (8.7%, 22.8%), *p <* 0.001
Gametocyte carriage^†^, percent (number positive/number sampled)	2.8% (20/707)	1.1% (8/717)	61% (−2.9%, 85%)	1.7% (0%, 3.4%), *p =* 0.05
Mean Hb, g/l^†^	101	107		5.9 (1.6, 10.2), *p =* 0.009
Anaemia (Hb < 110 g/l)^†^	63.1%	51.5%	18% (4.4%, 30%)	11.6% (3.0%, 20.2%), *p =* 0.010
Severe anaemia (Hb < 60 g/l)^†^, percent (number anaemic/number sampled)	2.7% (19/707)	1.3% (9/717)	53% (−21%, 82%)	1.4% (−0.25%, 3.1%), *p =* 0.093
Deaths from all causes, rate/1,000 (number of deaths/1,000s of person-months)*	1.0 (12/11.48)	1.1 (14/12.27)		−0.10 (−1.18, 0.99), *p =* 0.856

Children aged less than 10 years (aged 3–119 months at cycle 1). The coefficient of variation between villages after stratification was 0.37 for malaria incidence. The intra-class correlation for parasite prevalence was 0.045 and for mean Hb concentration was 0.06. *p*-values from tests for interaction between age group and intervention (tests of effect modification by age group): malaria incidence, *p =* 0.631; prevalence of parasitaemia, *p =* 0.187; gametocyte carriage, *p =* 0.360; mean Hb concentration, *p =* 0.797; anaemia, *p =* 0.237; severe anaemia, *p =* 0.191.

*Confirmed cases or deaths per 1,000 child-months at risk (number of cases or deaths/1,000s of child-months).

^†^Measured at the end of the transmission season.

CCM, community case management; Hb, haemoglobin; SMC, seasonal malaria chemoprevention.

**Table 3:** Efficacy and impact of SMC against malaria, parasitaemia, gametocyte carriage, anaemia, and deaths, in each age group.

Outcome	CCM	SMC+CCM	Efficacy (95% CI)	Difference^‡^ (95% CI)
**Children <5 years (aged 3–59 months at cycle 1)**				
Malaria cases, rate/1,000 (cases/1,000s of person-months)*	134.6 (797/5.92)	23.8 (145/6.10)	82% (71%, 89%)	110.8 (64.7, 156.8), *p <* 0.001
Prevalence of parasitaemia^†^	18%	5.7%	69% (42%, 83%)	12.5% (6.8%, 18.2%), *p <* 0.001
Gametocyte carriage^†^	3.5%	1.1%	69% (−2.1%, 91%)	2.45% (−0.6%, 5.5%), *p =* 0.105
Mean Hb, g/l^†^	94	101		7 (2, 11), *p =* 0.007
Anaemia (Hb < 110 g/l)^†^	77.4%	68.4%	12% (0.0%, 22%)	9.1% (0.6%, 17.5%), *p =* 0.037
Severe anaemia (Hb < 60 g/l)^†^	3.3%	0.81%	75% (13%, 93%)	2.5% (0.2%, 5.0%), *p =* 0.036
Deaths from all causes, rate/1,000 (deaths/1,000s of person-months)*	1.87 (11/5.92)	1.98 (12/6.10)		−0.11 (−2.0, 1.8)
**Children 5–9 years (aged 60–119 months at cycle 1)**				
Malaria cases, rate/1,000 (cases/1,000s of person-months)*	121.6 (675/5.55)	20.2 (125/6.17)	83% (76%, 89%)	101.3 (66.7, 136.0), *p <* 0.001
Prevalence of parasitaemia^†^	25%	5.8%	77% (53%, 89%)	19.3% (8.3%, 30.2%), *p =* 0.001
Gametocyte carriage^†^	2.1%	1.2%	44% (−115%, 86%)	0.9% (−1.0%, 2.8%), *p =* 0.327
Mean Hb, g/l^†^	109	114		5.2 (0.4, 9.9), *p =* 0.035
Anaemia (Hb < 110 g/l)^†^	47.5%	33.4%	30% (5.4%, 48%)	14.1% (3.1%, 25.0%), *p =* 0.014
Severe anaemia (Hb < 60 g/l)^†^	2.1%	1.7%	16% (−224%, 78%)	0.3% (−2.1%, 2.8%), *p =* 0.778
Deaths from all causes, rate/1,000 (deaths/1,000s of person-months)*	0.18 (1/5.55)	0.32 (2/6.17)		−0.14 (−0.66, 0.37)

^‡^Difference: CCM − SMC+CCM.

*Confirmed cases or deaths per 1,000 child-months (number of cases or deaths/1,000s of child-months) for July–November 2011.

^†^Measured at the end of the transmission season.

CCM, community case management; Hb, haemoglobin; SMC, seasonal malaria chemoprevention.

**Table 4:** Efficacy of SMC and the number of malaria cases averted per 1,000 children, in each month.

Month	Number of malaria cases/1,000/month		Efficacy (95% CI)	Rate difference per 1,000 (95% CI)	*p*-Value
	CCM	SMC+CCM			
1	203.9	25.5	87.5% (79.6%, 92.3%)	178.4 (121.3, 235.4)	<0.001
2	223.9	42.1	81.2% (69.1%, 88.5%)	181.8 (117.4, 246.2)	<0.001
3	162.9	34.5	78.8% (65.6%, 87.0%)	128.5 (72.7, 184.2)	<0.001
4	61.0	10.6	82.6% (59.5%, 92.5%)	50.3 (14.9, 85.8)	0.007
5	17.8	2.1	88.0% (61.6%, 96.3%)	15.6 (−0.1, 31.3)	0.051

CCM, community case management; SMC, seasonal malaria chemoprevention.

### The prevalence of *P*. *falciparum* parasitaemia and gametocyte carriage at the end of the transmission season

**Fig 7:**
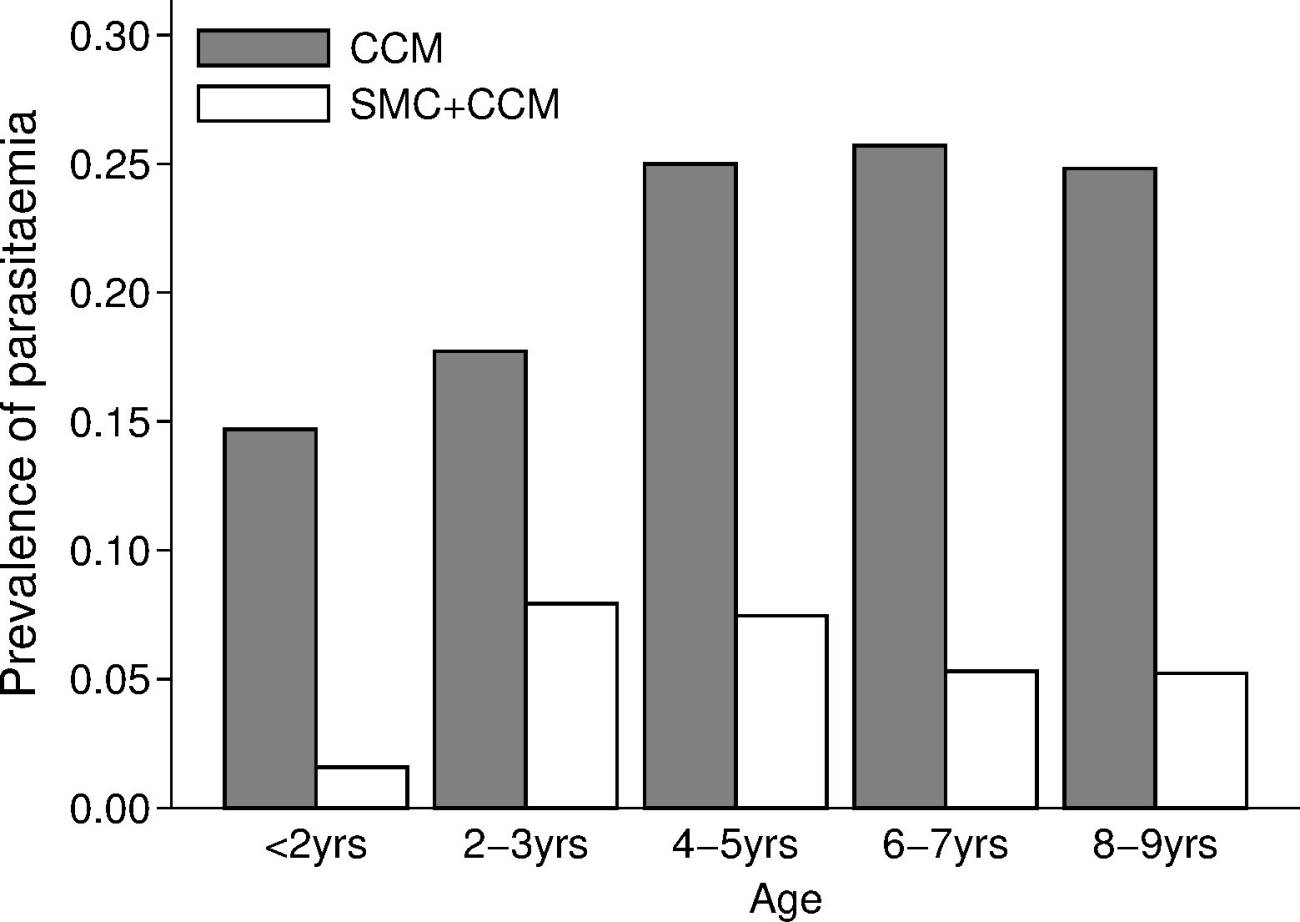
The effect of SMC on the prevalence of parasitaemia. The prevalence of *P*. *falciparum* infection at the end of the transmission season in each arm of the trial, by age group. CCM, community case management; SMC, seasonal malaria chemoprevention.

**Fig 8:**
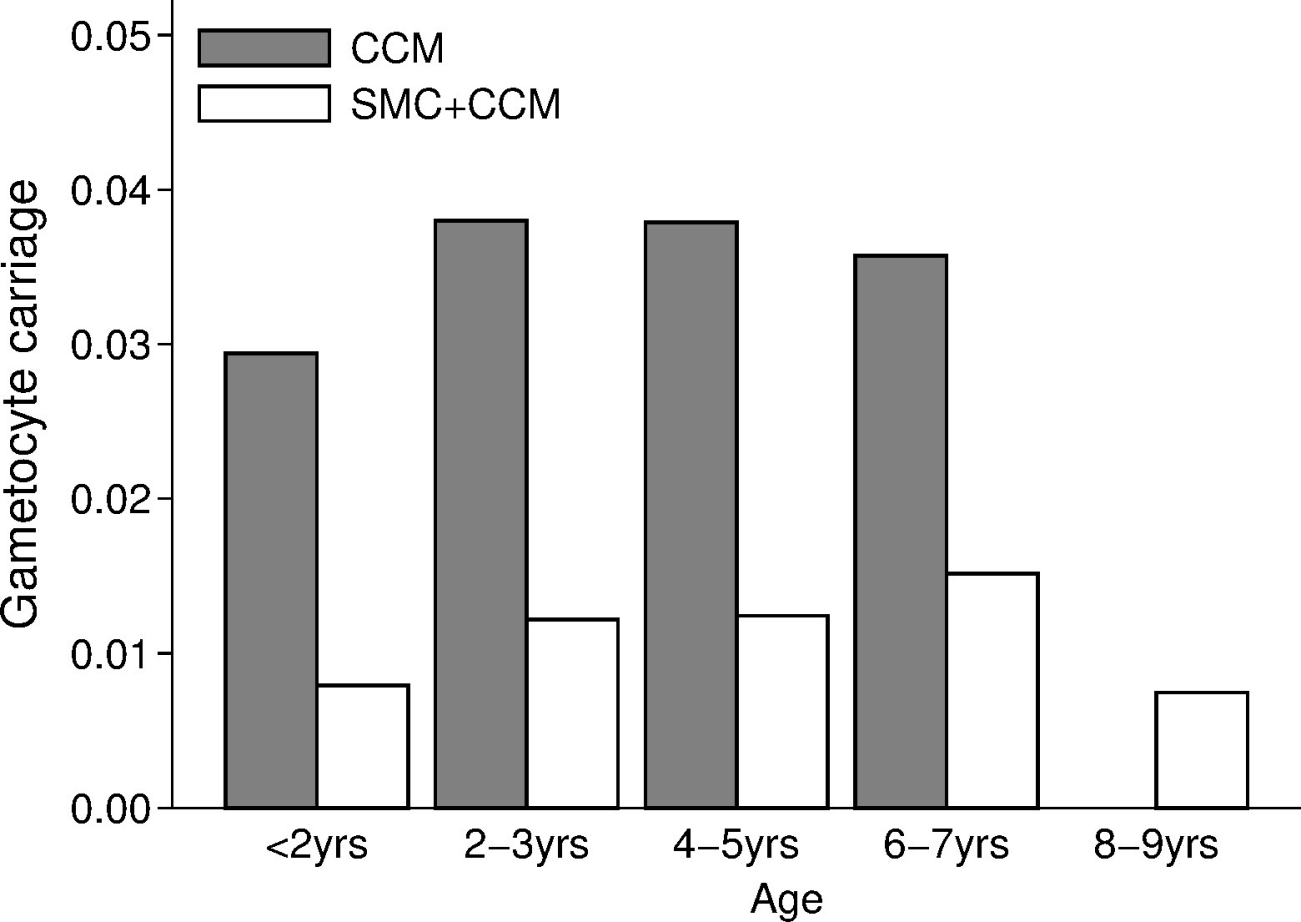
The effect of SMC on the prevalence of gametocyte carriage: The prevalence of gametocyte carriage at the end of the transmission season in each arm of the trial, by age group. There were no carriers aged 8–9 years in the CCM group. CCM, community case management; SMC, seasonal malaria chemoprevention.

The prevalence of asexual *P*. *falciparum* infection in control villages at the end of the transmission season increased with children’s age, from about 15% in children under 2 years of age to about 25% in children aged 4–9 years (Fig 7). The overall prevalence in the control villages was 21.5% (152/707 children surveyed positive by microscopy), compared to 5.7% (41/717) in the SMC villages, a reduction of 73% (95% CI 52%, 85%). The reduction associated with SMC was slightly greater in the age group 5–9 years (77%) compared to the group aged less than 5 years (69%) (*p*-value for interaction *p =* 0.187). Gametocyte carriage (Fig 8) was 2.8% (20/707 children) in control villages and 1.1% (8/717) in SMC villages, a reduction of 61% (95% CI −2.9%, 85%).

### The prevalence of anaemia at the end of the transmission season

**Fig 9:**
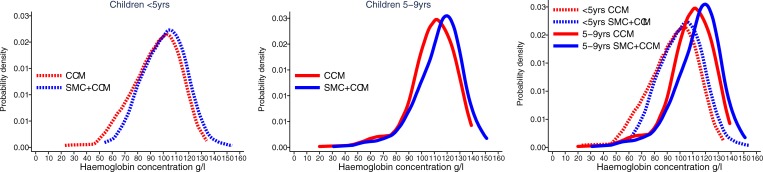
The effect of SMC on anaemia. The distribution of haemoglobin concentrations measured at the end of the transmission season is shown, for each age group of children, in villages with SMC and in control villages, using a kernel density estimator to plot the smoothed distribution of concentrations in each group. The left-hand plot shows children aged less than 5 years, the middle plot children 5–9 years of age. In the right-hand plot the 2 age groups have been superimposed for comparison. CCM, community case management; SMC, seasonal malaria chemoprevention.

The mean Hb concentration in children aged less than 5 years was 94 g/l in the control villages and 101 g/l in the SMC villages, an increase of 6.5 g/l (95% CI 2.0, 11) associated with SMC. In the older children, the mean Hb concentration was 109 g/l in the control villages and 114 g/l in the SMC villages, an increase of 5.2 g/l (95% CI 0.4, 9.9). A total of 19/707 (2.7%) children in the control villages had severe anaemia (Hb < 60 g/l), compared to 1.3% (9/717) in the SMC group. There was weak evidence that SMC was associated with a greater reduction in severe anaemia in the age group under 5 years (interaction *p*-value = 0.191). Among children aged less than 5 years, 3.3% had Hb < 60 g/l in the control villages compared to 0.81% in the SMC villages, a reduction of 75% (95% CI 13%, 93%). The distribution of Hb concentration in each age group and intervention group is shown in Fig 9.

### Adverse events

Twenty-six children were known to have died during the study: 12 deaths in the control villages were reported by village reporters, while in the SMC villages 8 deaths were reported by village reporters and a further 6 were detected when the child’s absence during SMC cycles was investigated by the study team. Twenty-three of the deaths were in children aged less than 5 years, and 3 in children 5–9 years of age. The mortality rate ratio (SMC:control) was 1.1 (95% CI 0.4, 3.0). For the 12 deaths in control villages, the cause of death at verbal autopsy was considered to be malaria (9 children), meningitis (1 child), pneumonia (1 child), or sepsis (1 child). For the 14 deaths in SMC villages, the cause of death was considered to be malaria (7 children), malaria and pneumonia (1 child), malaria and diarrhoea (1 child), diarrhoea (3 children), acute respiratory infection (1 child), or sepsis (1 child). Sixteen of the 26 deaths occurred at home, 8 occurred in the health post, 1 in the district health centre, and 1 while the child was being transported to the health centre. Of the 14 deaths in SMC villages, the exact date of death—and hence the timing of the death in relation to the start of the most recent course of SMC—was available for 6 children. One of these children (whose death was considered to be due to malaria and pneumonia) had not received SMC; one died 26 days after SMC (of malaria and diarrhoea), one 33 days after SMC (of diarrhoea), one 26 days after SMC (of malaria), one 27 days after SMC (of sepsis), and one 9 days after SMC (of diarrhoea).

A total of 29 cases of suspected adverse drug reactions were reported by CHWs. Vomiting was the most commonly reported symptom (11 cases). There was 1 case of rash on both arms and chest, occurring 3 days after the child received the first dose of SMC in cycle 1 (this child was excluded from further SMC). None of the adverse events was considered severe.

### Accuracy of malaria RDTs

Blood films from 1,126 patients with a positive RDT and from 227 patients with a negative RDT were examined by microscopy. RDT sensitivity, compared to microscopy, was 95%, and specificity was 48% (Table 5). There was some evidence that specificity was higher in the SMC villages (63% compared to 42% in CCM villages, *p =* 0.054) and sensitivity lower (79% compared to 97%, *p =* 0.037).

**Table 5:** Comparison of RDT results with microscopy, in a sample of RDT-positive and RDT-negative patients.

RDT outcome	CCM alone			SMC+CCM		
	Microscopy		Total	Microscopy		Total
	Positive	Negative		Positive	Negative	
RDT+	836	153	989	96	41	137
RDT−	22	111	133	25	69	94

CCM, community case management; RDT, rapid diagnostic test; SMC, seasonal malaria chemoprevention.

### The prevalence of molecular markers of resistance to SMC drugs among incident malaria cases

RDT-derived DNA from 240 incident malaria cases were typed, 110 from control villages and 130 from SMC villages (Table 6). About 40% of samples in each group carried both the *pfmdr1*-86Y and -184Y mutation (38% in the control villages and 42% in the SMC villages). The *pfdhfr* mutations (51I, 59R, and 108N) and *pfdhps*-437A occurred more frequently in the cases from the SMC villages (Table 6). The *pfdhps*-540E mutation was found in 2 samples, 1 from a control village and 1 from an SMC village. These 2 samples carried only the wild type at *pfdhps* codons 436, 437, and 613. Mutations at *pfmdr1*-1246 were not typed in the samples from malaria cases.

**Table 6:** Prevalence of molecular variants associated with resistance to sulfadoxine-pyrimethamine and amodiaquine, in samples from malaria cases.

Variant	CCM		SMC+CCM		Difference (95% CI)	*p*-Value
	Number positive/number typed	Prevalence (95% CI)	Number positive/number typed	Prevalence (95% CI)		
***pfmdr1*-86Y**	77/110	0.70 (0.55, 0.85)	76/123	0.62 (0.49, 0.74)	−0.08 (−0.28, 0.11)	0.394
***pfmdr1*-184Y**	57/110	0.52 (0.46, 0.57)	69/123	0.56 (0.50, 0.62)	0.04 (−0.04, 0.12)	0.280
***pfdhfr*-51I**	34/110	0.31 (0.19, 0.43)	54/130	0.42 (0.34, 0.49)	0.11 (−0.03, 0.25)	0.131
***pfdhfr*-59R**	28/110	0.25 (0.14, 0.36)	59/130	0.45 (0.34, 0.57)	0.20 (0.04, 0.36)	0.018
***pfdhfr*-108N**	28/110	0.25 (0.16, 0.35)	66/130	0.51 (0.38, 0.63)	0.25 (0.10, 0.41)	0.003
***pfdhps*-436A**	33/110	0.30 (0.18, 0.42)	59/130	0.45 (0.38, 0.53)	0.15 (0.02, 0.29)	0.031
***pfdhps*-437A**	43/110	0.39 (0.29, 0.49)	46/130	0.35 (0.29, 0.42)	−0.04 (−0.15, 0.08)	0.519
***pfdhps*-540E**	1/70	0.01 (−0.01, 0.04)	1/118	0.01 (−0.01, 0.02)	−0.01 (−0.04, 0.03)	0.721
***pfdhps*-613S**	2/70	0.03 (−0.01, 0.07)	3/118	0.03 (−0.01, 0.06)	0.00 (−0.05, 0.05)	0.899
***pfmdr1*-86Y+184Y**	42/110	0.38 (0.28, 0.48)	52/123	0.42 (0.35, 0.49)	0.04 (−0.08, 0.16)	0.486
*pfdhfr*-51I+59R+108N*	19/108	0.18 (0.08, 0.27)	48/127	0.38 (0.30, 0.46)	0.20 (0.08, 0.32)	0.003
***pfdhps* haplotypes 431, 436, 437, 540, 613^#^**						
IAAKAA	14/68	0.21 (0.10, 0.31)	26/113	0.23 (0.19, 0.27)	0.02 (−0.09, 0.14)	0.664
IAGKAA	9/68	0.13 (0.04, 0.22)	23/113	0.20 (0.11, 0.29)	0.07 (−0.06, 0.20)	0.254
IAGKAS	2/68	0.03 (−0.01, 0.07)	2/113	0.02 (0.00, 0.04)	−0.01 (−0.06, 0.03)	0.590
ISAKAA	10/68	0.15 (0.07, 0.22)	13/113	0.12 (0.05, 0.18)	−0.03 (−0.13, 0.07)	0.521
ISGEAA	1/68	0.01 (−0.02, 0.04)	1/113	0.01 (−0.01, 0.03)	−0.01 (−0.04, 0.03)	0.726
ISGKAA	37/68	0.54 (0.43, 0.66)	54/113	0.48 (0.40, 0.56)	−0.07 (−0.21, 0.07)	0.332
ISGKAS	0/68	0.00 (0.00, 0.00)	1/113	0.01 (−0.01, 0.03)	0.01 (−0.01, 0.03)	0.289

*Five samples mixed for 2 codons were excluded.

^#^Seven samples mixed for 2 codons and 52 samples with missing data were excluded.

CCM, community case management; SMC, seasonal malaria chemoprevention.

### The prevalence of molecular markers of resistance to SMC drugs among children surveyed at the end of the transmission season

In the survey at the end of the transmission season, 182 positive samples were typed, 140 from control villages and 42 from SMC villages (Table 7). The *pfmdr1*-86Y mutation was found in only 11 samples, and only 2 samples (both in the control group) carried both the 86Y and 184Y mutations. The *pfmdr1*-1246Y mutation was found in 1 sample, in the control group. This sample carried the 184Y mutation but not the 86Y mutation. The *pfdhfr* triple mutation 51I+59R+108N was more common in the end of season survey than among the clinical cases, but the prevalence was similar in both intervention groups (55% in the control group and 66% in the SMC group in the end of season survey, compared to 18% and 38%, respectively, in the clinical cases). The *pfdhps*-540 mutation was not identified in the survey samples.

**Table 7:** Prevalence of molecular variants associated with resistance to sulfadoxine-pyrimethamine and amodiaquine, from survey samples at the end of the transmission season.

Variant	CCM		SMC+CCM		Difference (95% CI)	*p*-Value
	Number positive/number typed	Prevalence (95% CI)	Number positive/number typed	Prevalence (95% CI)		
***pfmdr1*-86Y**	5/140	0.04 (0.00, 0.07)	6/42	0.14 (0.04, 0.24)	0.11 (0.00, 0.21)	0.044
***pfmdr1*-184Y**	96/140	0.69 (0.62, 0.75)	24/42	0.57 (0.49, 0.65)	−0.11 (−0.22, −0.01)	0.037
***pfmdr1*-1246Y**	1/140	0.01 (−0.01, 0.02)	0/42	0.00 (0.00, 0.00)	−0.01 (−0.02, 0.01)	0.297
***pfdhfr*-51I**	91/140	0.65 (0.56, 0.74)	21/30	0.70 (0.62, 0.78)	0.05 (−0.07, 0.17)	0.393
***pfdhfr*-59R**	92/140	0.66 (0.60, 0.72)	20/30	0.67 (0.53, 0.80)	0.01 (−0.14, 0.16)	0.895
***pfdhfr*-108N**	100/140	0.71 (0.65, 0.77)	22/30	0.73 (0.63, 0.84)	0.02 (−0.10, 0.14)	0.738
***pfdhps*-436A**	45/140	0.32 (0.22, 0.42)	11/42	0.26 (0.11, 0.41)	−0.06 (−0.24, 0.12)	0.505
***pfdhps*-437A**	65/140	0.46 (0.34, 0.59)	19/42	0.45 (0.22, 0.68)	−0.01 (−0.27, 0.25)	0.926
***pfdhps*-613S**	3/140	0.02 (0.00, 0.05)	4/42	0.10 (0.04, 0.15)	0.07 (0.02, 0.13)	0.016
***pfmdr1* haplotypes 86, 184, 1246**						
YYD	2/139	0.01 (0.00, 0.03)	0/42	0.00 (0.00, 0.00)	−0.01 (−0.03, 0.01)	0.148
YND	3/139	0.02 (0.00, 0.05)	6/42	0.14 (0.04, 0.24)	0.12 (0.02, 0.22)	0.024
NYY	1/139	0.01 (0.00, 0.02)	0/42	0.00 (0.00, 0.00)	−0.01 (−0.02, 0.01)	0.296
NYD	93/139	0.67 (0.60, 0.73)	24/42	0.57 (0.49, 0.65)	−0.10 (−0.20, 0.01)	0.067
NND	47/139	0.34 (0.27, 0.41)	13/42	0.31 (0.20, 0.42)	−0.03 (−0.16, 0.10)	0.658
***pfdhfr*-51I+59R+108N**	68/123	0.55 (0.46, 0.64)	19/29	0.66 (0.52, 0.79)	0.10 (−0.06, 0.26)	0.194
***pfdhps* haplotypes 431, 436, 437, 540, 581, 613**						
IAAKAA	28/132	0.21 (0.14, 0.28)	6/42	0.17 (0.06, 0.27)	−0.05 (−0.17, 0.08)	0.467
IAGKAA	8/132	0.06 (0.02, 0.10)	1/42	0.02 (−0.02, 0.07)	−0.04 (−0.10, 0.03)	0.239
IAGKAS	1/132	0.01 (−0.01, 0.02)	3/42	0.07 (0.01, 0.14)	0.06 (0.00, 0.13)	0.062
ISAKAA	34/132	0.26 (0.17, 0.35)	12/42	0.29 (0.13, 0.45)	0.03 (−0.16, 0.21)	0.753
ISGKAA	72/132	0.55 (0.42, 0.67)	19/42	0.45 (0.28, 0.63)	−0.09 (−0.31, 0.13)	0.386
ISGKAS	1/132	0.01 (−0.01, 0.02)	1/42	0.02 (−0.02, 0.07)	0.02 (−0.03, 0.06)	0.469

CCM, community case management; SMC, seasonal malaria chemoprevention.

## Discussion

In this cluster randomised trial of SMC with CCM for malaria in children aged less than 10 years delivered over 5 months in the Saraya district of south-east Senegal, we showed that SMC was associated with a reduction of more than 100 malaria cases per 1,000 children per month both in children aged less than 5 years of age and in children aged 5–9 years, compared to CCM alone, and with a reduction in the prevalence of anaemia and malaria parasitaemia at the end of the transmission season in both age groups. SMC was well tolerated over this period, which is longer than studied previously [3] and longer than is currently used in national SMC programmes, where SMC is provided for 3 or 4 months [1]. A relatively small reduction in the number of cases was shown in the fifth month of treatment, but because incidence was already very high at the time of the first cycle, it is possible that if cycle 1 had started earlier, in early July, the fifth treatment might have had more impact. Delivery of SMC by resident CHWs, who were also providing CCM for malaria, achieved high coverage each month and ensured that children with symptomatic malaria were promptly treated with artemether-lumefantrine.

The findings of this study are consistent with those of Cissé et al. [3], who showed that SMC was effective in children aged less than 10 years. That study was done in an area with a shorter transmission season and lower transmission intensity than the present study. Our study shows that in an area with more intense malaria transmission, with a significant burden of malaria in older children, these children benefitted from being included in SMC. In the previous study, SMC given to children up to the age of 10 years was associated with a reduction in transmission, as shown by reduced incidence in those who did not receive SMC (children above the age for SMC and adults) in the areas where SMC was used. In our study we were not able to measure indirect effects on transmission, but it is likely that indirect effects contributed to the results of our study. The relative magnitude of indirect effects may be expected to be somewhat lower in areas of higher transmission intensity.

Several previous studies have combined intermittent preventive treatment and CCM for malaria [11–14]. Two studies in Ghana—a pilot study [11] and a cluster randomised trial [12]—evaluated intermittent preventive treatment of children aged less than 5 years combined with presumptive treatment of fever cases and showed that the combined strategy reduced the number of presumptive cases. A study in The Gambia showed that village health workers could successfully combine testing and treatment of malaria with administration of SMC, but the malaria incidence was too low to determine any impact of SMC [13]. In a pilot study in Senegal, CHWs providing home management for malaria were trained to deliver SMC for 2 months; this study showed a reduction in malaria incidence associated with SMC and a reduction in prevalence at the end of the transmission season [14].

Combining SMC delivery with CCM increases the promptness of treatment of breakthrough cases, and has other potential advantages including opportunities for catch-up of missed SMC doses and reduced costs of delivery for both interventions [7]. During SMC cycles, children who are unwell are not given SMC drugs but are referred for testing with an RDT so that they can be given ACT if they have malaria [1]. In villages without access to testing and treatment for malaria locally, children found to be unwell during SMC visits are referred to the nearest health facility. In Mali, SMC has been delivered in communities at fixed points by mobile teams able to administer SMC and to test febrile children with an RDT and treat with an ACT if the test was positive [15]. It is not known whether this approach can be taken door to door effectively.

Our study had a number of limitations. One limitation was that we were not able to show an impact of SMC on mortality or on severe malaria. The study was not designed to measure an effect on mortality, and it is possible that some deaths may have been missed because additional deaths not reported by village reporters were detected in the SMC arm of the trial when children who were absent at SMC cycles were investigated. We collected data on all admissions to Saraya Hospital during the year of the study in order to determine the age distribution and seasonal pattern of malaria hospital admissions. These data showed that there was a similar number of malaria admissions in the group aged 5–9 years as in the age group under 5 years, the majority occurring during the 5 months from July to November, suggesting that SMC targeting children under the age of 10 years for 5 months might be needed. In the control villages in our study, 17% of children had malaria on 2 or more occasions, and 3 children had malaria 5 times in 5 months, while only 0.8% of children in SMC villages had more than 1 malaria episode. Children who have more episodes of clinical malaria are more likely to develop severe disease that leads to hospital admission [16]. Although we could not demonstrate an impact of SMC on severe disease, the impact on uncomplicated malaria suggests that the intervention might also be effective in preventing severe cases, as has been observed in earlier trials [17,18].

Another limitation of the study was that we were not able to obtain malaria smears for all malaria cases: CHWs had been trained to make blood films but not all were able to do this consistently. Parasitological confirmation of malaria therefore relied on RDTs, which may have resulted in false positives. Although the study was not designed to measure RDT characteristics, we noted that when the accuracy of diagnosis by RDT was compared with microscopy, specificity was low, as has been observed in other high-transmission settings [19] due to persistence of HRP2 protein. Specificity was higher in the SMC villages, reflecting the lower exposure to infection. RDT sensitivity was somewhat lower in the SMC villages, possibly due to lower parasite densities.

SMC was well tolerated; however, we were not able to monitor effects of repeated treatments on biochemical parameters and haematological parameters (other than Hb concentration at the end of the transmission season).

CCM linked to SMC ensures that breakthrough malaria cases can be promptly treated with artemether-lumefantrine. We found that the frequency of the *pfmdr1* mutations 86Y and 184Y, associated with AQ resistance, was lower in children carrying *P*. *falciparum* parasites at the end of the transmission season than in samples taken from children presenting with malaria during the study. It is known that treatment with artemether-lumefantrine selects for wild-type alleles at these loci [20]. Prompt treatment of malaria cases in SMC areas may therefore act to limit selection for AQ resistance by SMC.

Our results suggest that the impact of SMC programmes could be substantially increased by including older children. The detailed distribution of malaria cases by age is often not available to health managers, due to the fact that reporting of malaria cases to national health management information systems is in broad age groups (under 5 years, and 5 years and above; in some countries, 3 categories are used: under 5 years, 5–14 years, and 15 years and above). Individual patient data may need to be obtained from hospitals to determine the appropriate upper age limit for SMC programmes. SMC was well tolerated in our study, but further studies should assess the safety of repeated SMC treatments if given for longer periods. SMC can act as a screening programme, but in areas with poor access to healthcare, referral of sick children may be problematic. Combining SMC with CCM services in such areas should be further evaluated. Other household members could also be screened for malaria, especially slightly older children who have stopped receiving SMC.

This cluster randomised trial of SMC in children aged less than 10 years in a high-transmission area in the south-east of Senegal showed that high coverage of SMC could be achieved over 5 months and that the treatments were well tolerated, were associated with a similar number of reductions in malaria episodes in the age groups under 5 years and 5–9 years, and reduced the prevalence of parasitaemia and anaemia in both age groups. Combining SMC with CCM ensured children with malaria were promptly treated with artemether-lumefantrine.

Twelve countries now have SMC programmes, reaching about 17 million children in 2017 [21]. Despite the challenges of delivering SMC, many countries have achieved high coverage and have seen substantial reductions in malaria cases. This success has led national malaria control programmes to consider whether older children should be included in SMC programmes, and whether more than 4 SMC cycles can be given, which would allow expansion of the geographical area targeted by SMC programmes to include communities living in areas where the transmission season is longer. The findings of this study indicate that SMC could be administered over a longer period and/or to a wider age group. The SMC strategy used in this study could be adapted, based on local epidemiology, to increase the impact of SMC on malaria.

## Supporting information

S1 French Abstract French translation of the abstract by JLN. (DOCX)

## Abbreviations

ACT: artemisinin combination therapy
AQ: amodiaquine
ASC: *agent de santé communautaire*
CCM: community case management
CHW: community health worker
DSDOM: *distributeur de soins à domicile*
Hb: haemoglobin
RDT: rapid diagnostic test
SMC: seasonal malaria chemoprevention
SP: sulfadoxine-pyrimethamine
