# Supplementary material for: Seasonal malaria chemoprevention combined with community case management of malaria in children under 10 years of age, over 5 months, in south-east Senegal: A cluster-randomised trial
Source: PLoS Med. 2019 Mar 13;16(3):e1002762. doi: 10.1371/journal.pmed.1002762 (PMC6415816; doi:10.1371/journal.pmed.1002762)
Supplement: S1 French Abstract — (DOCX) [file pmed.1002762.s001.docx]

Chimioprévention du paludisme saisonnier combinée à la prise en charge communautaire du paludisme chez les enfants de moins de 10 ans pendant 5 mois dans le sud du Sénégal: essai clinique randomisé en grappes.

Abstract

Contexte:

La chimioprévention du paludisme saisonnier (CPS) est recommandée dans le sahel chez les enfants de moins de 5 ans, pendant 4 mois. Il peut être approprié d’inclure des enfants plus âgés et de les protéger plus de 4 mois. Nous avons évalué l'efficacité de la CPS avec la sulfadoxine-pyriméthamine plus amodiaquine pendant 5 mois chez les enfants de moins de 10 ans dans le district de Saraya au sud du Sénégal en 2011.

Méthodes et résultats:

Vingt-quatre villages avec 2301 enfants âgés de 3 à 59 mois et 2 245 âgés de 5 à 9 ans, ont été randomisés pour recevoir la CPS et/ou une prise en charge communautaire du paludisme (PECC). Dans tous les villages, des agents de santé communautaires (ASC) ont été formés pour traiter les cas de paludisme avec une combinaison à base de dérivés d’artémisinine après un test de diagnostic rapide (TDR). Dans les villages CPS, les ASC ont administré la CPS aux enfants âgés de 3 mois à 9 ans, une fois par mois pendant 5 mois. Les enfants ont été suivis de juillet à décembre. Le critère d'évaluation principal était le paludisme (fièvre ou antécédents de fièvre avec un TDR positif). La prévalence de l'anémie et de la parasitémie ont été mesurées lors d'une enquête transversale à la fin de la saison de transmission. Les marqueurs moléculaires de résistance du Plasmodium aux médicaments de la CPS ont été analysés chez les cas incidents de paludisme et chez des enfants porteurs asymptomatiques pendant l’enquête. La CPS a été bien tolérée avec aucun évènement indésirable noté. Il y a eu 1470 cas de paludisme confirmés par TDR dans les villages témoins et 270 dans les villages CPS. La différence de taux chez les enfants de moins de 5 ans était de 110,8 (IC 95% : 64,7- 156,8; p <0,001) et chez les enfants de 5 à 9 ans de 101,3 /1000 /mois (IC 95%: 66,7 -136,0; p <0,001). Le taux moyen d'hémoglobine était plus élevée dans les villages CPS que dans les villages témoins, de 0,65 g/dl (IC 95%: 0,20- 1,1; p = 0,007) chez les enfants de moins de 5 ans et de 0,52 g/dL. (IC 95% 0,04-0,99; p = 0,035) chez les enfants de 5 à 9 ans. La prévalence de la parasitémie était de 18% chez les enfants de moins de 5 ans et 25% chez les enfants de 5 à 9 ans dans les villages témoins, et de 5,7% et 5,8% respectivement dans ces deux groupes d'âge dans les villages CPS, une différence de prévalence de 12,5% (IC 95%: 6,8%- 18,2%; p <0,001) chez les moins de 5 ans et de 19,3% (IC 95%: 8,3%-30,2%; p <0,001) chez les 5 à 9 ans. La mutation Pfdhps-540E associée à une résistance clinique à la sulfadoxine-pyriméthamine a été retrouvée dans 0,8% des cas incidents de paludisme, mais non retrouvée lors de l'enquête. Douze enfants sont décédés dans le groupe témoin et 14 dans le groupe CPS, soit une différence de taux de 0,096/ 1 000 enfants-mois, IC à 95%: 0,99-1,18; p = 0,895. Une limite a été que nous n'étions pas en mesure d'obtenir des frottis sanguins pour tous les cas suspects de paludisme, et que nous nous en remettions aux TDR pour le diagnostic, lesquels pouvaient inclure des faux positifs.

Conclusion: La CPS chez les enfants de moins de 10 ans pendant 5 mois a été faisable, bien tolérée, très efficace pour prévenir les accès palustres et réduire la prévalence de la parasitémie et de l'anémie. La CPS combinée à la PEEC a obtenu une couverture élevée, assurant que les enfants souffrant d’accès palustres soient rapidement traités avec de l'artéméther-luméfantrine et réduisant ainsi la charge de travail des ASC.
